# Effect of non-surgical periodontal treatment on three salivary adipokines in diabetic patients with periodontitis

**DOI:** 10.34172/joddd.2020.029

**Published:** 2020-09-21

**Authors:** Narges Ziaei, Shima Golmohammadi, Mari Ataee, Farnoosh Ardalani, Mehran Mesgari Abbasi

**Affiliations:** ^1^Department of Periodontics, Dental School, Kermanshah University of Medical Sciences, Kermanshah, Iran; ^2^Department of Periodontics, Dental School, Islamic Azad University of Borujerd, Borujerd, Iran; ^3^Faculty of Medicine, Kermanshah University of Medical Sciences, Kermanshah, Iran; ^4^Dentist, Private Practice, Kermanshah, Iran; ^5^Researcher, Drug Applied Research Center, Tabriz University of Medical Sciences, Tabriz, Iran

**Keywords:** Adipokines, Human chemerin protein, Non-surgical periodontal debridement, Periodontitis, progranulin, Type 2 diabetes mellitus, Visfatin

## Abstract

**Background.** This study investigated the effect of non-surgical periodontal treatment on clinical indices and salivary levels of visfatin, chemerin, and progranulin in diabetic patients with periodontitis.

**Methods.** This interventional clinical trial was performed on 20 patients with type II diabetes mellitus (T2DM) with moderate to severe chronic periodontitis (periodontitis stages II or III according to the new classification of periodontal diseases). Clinical indices, including gingival index (GI), probing pocket depth (PPD), clinical attachment level (CAL) and plaque index (PI), were recorded and visfatin, chemerin, and progranulin adipokines levels were also measured in unstimulated saliva by ELISA technique at baseline and twelve weeks after non-surgical periodontal treatment.

**Results.** GI dropped from 1.92±0.27 to 0.71±0.14 after the intervention (P<0.001). Also, there were significant changes in the PPD and PI (P<0.001). However, no significant changes were observed in the CAL (P<0.05). The concentrations of all three salivary adipokines decreased after treatment, but this change was statistically significant only for progranulin (P<0.05).

**Conclusion.** Non-surgical periodontal therapy resulted in improvements in the clinical indices of GI, PPD, and PI in T2DM patients with periodontitis. Moreover, the significant reduction in the salivary level of progranulin after treatment suggests that it might be considered a target inflammatory marker in periodontal diseases.

## Introduction

Periodontitis is a local inflammatory disease of the tooth-supporting tissues, characterized by gingival bleeding, loss of alveolar bone, and attachment loss.^1^ Although microorganisms are the main etiologic agents triggering periodontal destruction, the host’s inflammatory chemical mediators play a central role in this respect.^2^ The proinflammatory cytokines produced locally as a reaction to the bacterial plaque^3^ probably affect the concentration of proinflammatory biomarkers in the plasma.^4^ Thus, periodontitis might have an even more significant impact on the systemic inflammatory condition in individuals with diabetes, possibly affecting the metabolic state of individuals with diabetes.^5^ That is why current research has shown a particular interest in periodontal diseases while analyzing the risk factors of various diseases.^6,7^

On the other hand, diabetes is a known risk factor for periodontal diseases and results in the increased destruction of periodontium.^8^ Studies have revealed that if diabetes is not diagnosed or is poorly controlled, periodontal therapies will not be very successful.^9^

The clinical and pathogenic association between periodontitis and general health and systemic diseases, like diabetes, has been the subject of many investigations during the past decade.^10^ Based on the evidence, the mutual interaction of these two illnesses could be related to the secretion of inflammatory mediators and proinflammatory cytokines, each with a key role in both diseases.^11^ It is believed that periodontal therapy can theoretically lead to reduced systemic inflammation and can exert its control over diabetes via common pathways of pathogenesis.^12-14^

The treatment of periodontitis focuses on terminating the destruction of the periodontium, usually through non-surgical removal of pathogenic bacteria in the periodontal pocket (i.e., scaling and mechanical debridement of the root surface).^15,16^ Non-surgical treatment of periodontitis results in reduced probing pocket depth, improved plaque and bleeding indices, increased surface for gingival adhesion, and reduced inflammation.^17^ Successful periodontal treatment might also reduce the morbidity and mortality rates of systemic diseases.^18^

Although the current standard procedure in the examination and treatment of periodontitis is based on clinical and radiographic measures, studying the biomarkers might provide supplementary information on its pathogenesis.^19^ Oral ﬂuid biomarkers have recently been introduced as a complementary method to traditional techniques for detecting periodontitis in its early stages and assessing the risk of its progression, and monitoring the effects of periodontal treatments.^19,20^ Saliva is the most easily and non-invasively collected oral fluid that contains local and systemic biomarkers of periodontal disease, and it can be used as a helpful diagnostic tool.^21^ Moreover, salivary samples include a whole collection of fluids secreted from all the oral sites of periodontal tissues and are beneficial in the overall assessment of diseases.^22^

Adipokines are hormone-like proteins that play a pivotal role in the immune-inflammatory process.^23^ According to the evidence, some of them are engaged in the progression of periodontitis.^24^ Adipokines increase in patients with periodontitis. Besides, they interfere with the mechanism of type II diabetes.^25^ Therefore, salivary visfatin, chemerin, and progranulin can be analyzed as possible biologic mediators linking these two diseases.

Visfatin, which is also known as pre-B cell colony enhancing factor (PBEF)/nicotinamide phosphoribosyltransferase (NAMPT), is an adipokine produced in adipose tissue, macrophages, granulocytes, and monocytes.^26^ This pleiotropic mediator has antiapoptotic activity and activates B cells to exert immunoregulatory and proinflammatory effects in inflammation.^27^ According to Pradeep et al^12,28^ visfatin levels in the saliva, serum, and gingival crevicular fluid (GCF) increase in periodontitis compared to healthy individuals. They also stated that non-surgical periodontal therapy decrease high visfatin concentrations in GCF and serum of periodontal patients.^29^

Chemerin is another adipokine that is also identified as tazarotene-induced gene 2 and retinoic acid receptor responder 2 (RARRES2) and is secreted from adipose tissue, epithelium, endothelium fibroblasts, and keratinocytes.^30^ Chemerin is found in inflamed tissues since it functions as a chemo-attractant on binding to its specific receptors, namely ChemR23, and causes immature dendritic cells, monocytes, and macrophages to migrate to the inflammation site.^31^

Progranulin, which is also recognized as acrogranin, granulin or proepithelin, is an adipokine released from epithelial cells, neurons, immune system cells, and chondrocytes.^32^ It is released from ameloblasts, odontoblasts, and osteoblasts and plays a crucial role in the early stages of embryogenesis, odontogenesis, and amelogenesis.^33^ Progranulin has been linked with abdominal obesity, increased plasma glucose, and lipid disorders,^34^ and is involved in the wound healing, inflammation, and tissue defense.^33^

Özcan et al^35^ showed that the amount of visfatin, chemerin, and progranulin differs with various statuses of periodontal health, but no study has explored the possible changes of these three adipokines after non-surgical periodontal therapy in diabetes patients with chronic periodontitis. To the best of our knowledge, other studies exploring the effect of periodontal treatment on adipokines have focused only on visfatin.^24,29^ Bearing this in mind, the present study was designed to assess salivary levels of visfatin, chemerin, and progranulin in diabetic patients with periodontitis and determine the effect of non-surgical therapy on salivary levels of these three adipokines.

## Methods

### Participants

Twenty T2DM patients with chronic periodontitis were recruited from the Diabetes Center of Kermanshah for this interventional clinical trial. The research protocol was approved by the Ethics Committee of Kermanshah University of Medical Sciences (IR.kums.rec.1395.564). The study aims and methods were described to all the participants, and their written consent was obtained.

According to inclusion criteria, men and women with an age range of 20‒60 years were eligible if they had physician-diagnosed T2DM over three months, HbA1c<8 at screening, stability in the type and use of diabetic drugs three months prior to and during the study, absence of any physiologic dysfunctions affecting type II diabetes and periodontal disease. The subjects taking oral hypoglycemic drugs (either monotherapy or a combination) were enrolled, who achieved proper control of diabetes and stability of type and dosage of glycemic control drugs in order to assure certain homogeneity of the study group in terms of an underlying systemic condition. The patients treated by insulin were excluded. All the subjects who participated in this investigation had moderate to severe periodontitis (stages II or III according to the new classification of periodontal and peri-implant diseases and conditions in 2017).^36^ Radiographs were taken to confirm the diagnosis. The patients had at least 16 natural teeth and had not received any periodontal treatment in the previous six months. Individuals needing extensive restorative procedures and root canal therapy or the ones suffering from oral infection were excluded. Other exclusion criteria consisted of smoking (current or former smokers for <5 years), limited life expectancy, any emergency related to diabetes within 30 days, BMI (body mass index = weight/height^2^) <18.5 or >30, pregnancy or becoming pregnant during the study and use of antibiotics, NSAIDs, and immunosuppressive medications in the previous three months.

Clinical Assessment and Periodontal Treatment

A calibrated examiner performed periodontal examinations with a UNC probe (Hu-Friedy^®^ Manufacturing Inc.). The measurements were recorded by a calibrated periodontist, in four points per tooth for all the teeth except the third molars. The diagnosis of periodontitis was established based on the clinical parameters of clinical attachment level (CAL), gingival index (GI),^37^ and probing pocket depth (PPD). Parallel periapical radiographs were used to determine the presence of bone resorption. O’Leary plaque index (PI)^38^ was documented before periodontal treatment and three months after that to check the efficacy of instructions in achieving and maintaining oral hygiene.

Periodontal treatment comprising scaling and root planing (SRP) with ultrasonic (Varios 350, NSK Japan) and hand devices (Gracey curettes, Hu-Friedy^®^ Manufacturing Inc.) was carried out. The treatment was completed in two sessions in 100 minutes, within two weeks, from the patient’s initial visit. Oral hygiene instructions, including the use of interdental cleaning aids (e.g., dental floss and proximal brushes), were provided twice for each patient and were exactly demonstrated on a dental model.

Salivary Sample Collection and Biochemical Assay

Salivary samples were collected at baseline and three months after the periodontal treatment was completed. In each phase, 5 mL of unstimulated whole saliva was collected under similar conditions by spitting method from 9:00 to 11:00 am. The participants were asked not to drink or eat anything and not brush their teeth one hour before sampling. Moreover, the periodontal measurements were made at least one hour ahead of sampling so that the samples were not contaminated with blood. The collected samples were kept in sterile containers at -80°C before being defrosted for biochemical analysis. The saliva samples were cleared by centrifugation at 10000 g for five minutes. The supernatants were transferred to enzyme-linked immunosorbent assay (ELISA) kits to measure the amount of three salivary adipokines according to the manufacturer’s protocol for visfatin (Biovendor Research & Diagnostic Products: USA), chemerin (Biovendor Research & Diagnostic Products: USA), and progranulin (Biovendor Research & Diagnostic Products: USA). The results were expressed as ng/mL for the concentration of each adipokine.

### Statistical Analysis

Data were analyzed by SPSS 16. The normality of data was tested by the Kolmogorov-Smirnov test (P>0.05). Given the normality of data, paired t-test was applied to compare GI, PPD, CAL, and PI at baseline and three months after the intervention. The relationships between the laboratory and clinical parameters were evaluated using the Spearman rank test. P<0.05 was considered statistically significant. In order to determine the required sample size, a pilot study was conducted. The standard deviation of the adipokine level was calculated at 1.34 ng/mL. The sample size was calculated to detect a difference of 1.34 ng/mL between values of adipokines before and after intervention at the 0.05 probability level with a power of 80%. According to the power analysis, the required sample size was a minimum of 20 patients.

## Results

The subjects consisted of seven men (35%) and 13 women (65%) with a mean age of 51.85±6.42 years, ranging from 33 to 65. The participants had diabetes for 8±4.6 years on average.

The mean ± SD of BMI was 26.8±1.5 at baseline and 26.5±1.6 after treatment, with no significant change after treatment (P>0.05).

Table 1 shows the clinical indices at baseline and three months after treatment. Following non-surgical periodontal therapy, all the clinical indices improved. Except for the CAL, changes in clinical parameters were statistically significant. (P<0.05) No adverse effect of the periodontal treatment was observed in patients.

**Table 1:** Mean ± SD values for GI, PPD, CAL, and PI at baseline and three months after periodontal therapy in patients with type 2 diabetes with periodontitis

**Clinical parameters**	**Baseline**	**3 months**	**P-value**
**GI**	1.92 ± 0.27	0.71 ± 0.14	<0.001
**PPD (mm)**	4.53 ± 0.67	2.18 ± 0.45	<0.001
**CAL (mm)**	3.56 ± 0.5	3.29 ± 0.45	>0.05
**PI (%)**	72.6 ± 16.45	25.3 ± 2.8	<0.001

As shown in Table 2, non-surgical periodontal treatment reduced all the assessed adipokines; however, the change was significant only for progranulin (P=0.047).

**Table 2:** Mean ± SD values (ng/mL) of visfatin, chemerin, and progranulin at baseline and three months after periodontal therapy in patients with type 2 diabetes with periodontitis

**Markers in saliva**	**Baseline**	**3 months**	**P-value**
**Visfatin**	10.375 ± 6.491	8.325 ± 5.150	0.221
**Chemerin**	0.295 ± 0.147	0.247 ± 0.149	0.286
**Progranulin**	35.562 ± 7.437	26.830 ± 5.876	0.047

The correlations between salivary adipokines and the clinical periodontal indices are given in Table 3. Visfatin level was positively correlated with GI, and chemerin level showed a positive correlation with PPD, whereas progranulin had a positive correlation with GI and PPD. There was no correlation between salivary adipokine levels and age and BMI (P>0.05).

**Table 3:** The correlations of the laboratory parameters (visfatin, chemerin, and progranulin) and the clinical parameters (GI, PPD, CAL, and PI)

**Parameters**		**GI**	**PPD**	**CAL**	**PI**
**Visfatin**	r	0.296	0.125	0.499	0.514
	p	0.013*	0.305	0.208	0.157
**Chemerin**	r	0.355	0.293	0.086	0.132
	p	0.349	0.013*	0.490	0.278
**Progranulin**	r	0.245	0.300	0.187	0.212
	p	0.038*	0.014*	0.130	0.074

*Correlation is significant at the 0.05 level.

## Discussion

Non-surgical periodontal treatment can improve the status of patients with diabetes. This therapeutic advantage becomes much more critical when diabetic patients have no indications for surgical intervention. In a meta-analysis, Sgolastra et al^13^ reported that adipokines play a fundamental role in the pathogenesis of periodontitis in diabetic patients and supported the theory that periodontal therapy can improve diabetes control. This study aimed to evaluate the effect of conventional phase I periodontal treatment on the salivary levels of visfatin, chemerin, and progranulin in diabetic patients with periodontitis. These adipokines function as immunoregulatory agents in chronic inflammatory diseases.^35^ It has been shown that saliva is a favorable diagnostic substitute to serum for the analysis of inflammatory biomarkers since salivary samples are easily collected, non-invasive, and cost-effective.^39,40^ The adipokine content of saliva is either produced locally in oral tissues or is actively transported from the serum; thus, it might reflect the serum’s content.^40^ Furthermore, the composition of saliva in terms of biochemical molecules slightly changes during different periodontal health statuses.^35^

This study’s findings showed significant reductions in GI, PPD, and PI after periodontal treatment (P<0.001). However, CAL exhibited no significant change (P>0.05). The overall enhancement of clinical measures after non-surgical periodontal therapy has been reported in several studies.^41,42^ It seems that in systemic diseases like diabetes mellitus, the concentration of salivary adipokines undergo changes, through which it might be possible to find out the relationship between diabetes and periodontitis.^24,43^

Serum and salivary concentrations of adipokines depend on gender and age.^44^ A noticeable consideration regarding the advantage of salivary samples over serum samples is that previous studies have shown that BMI does not affect the concentrations of adipokines in saliva, while BMI affects their serum concentrations.^40,45^ However, in the current research, a BMI of 18.5‒30 was considered an inclusion criterion for the samples, and the mean BMI of patients was stable during the study period.

Visfatin has gained more attention than the other two adipokines, and its level in GCF, serum, and saliva has been widely studied. Özcan et al^43^ and Tabari et al^45^ showed that the salivary level of visfatin declines as an outcome of non-surgical periodontal treatment in systemically healthy individuals. However, we did not detect any significant difference between the visfatin level before and after SRP in unstimulated salivary samples of diabetic patients (P=0.221) Raghavendra et al^29^ reported higher concentrations of serum and GCF visfatin in patients with periodontitis than those with healthy periodontium, and visfatin concentration decreased after SRP treatment and reached the level similar to that of the healthy subjects.

The results of the present study are not consistent with those reported by Wu et al,^24^ who showed a significant reduction in visfatin concentration in the serum and GCF of periodontitis patients with T2DM.

In a study by Özcan et al^35^ visfatin was introduced as a proper inflammatory marker considering the higher level of visfatin in periodontitis and gingivitis compared to the healthy periodontium. However, the absence of difference between periodontitis and gingivitis shows that visfatin has low sensitivity for the degree of tissue destruction. Besides, Pradeep et al^12^ indicated that visfatin level in serum and GCF of diabetic patients with periodontitis was higher than those with healthy periodontium. Thus, visfatin level could potentially be considered a target marker for the analysis of interconnected chronic inflammatory conditions (i.e., diabetes and periodontitis) and assessment of responses to therapy. However, in the present study, the reduction in salivary visfatin level following treatment and the resolution of periodontal inflammation did not reach a statistically significant level to validate this presumption.

This study was the first to investigate the salivary levels of three previously mentioned adipokines in response to periodontal treatment since we included periodontitis patients with diabetes. Chemerin is an adipocytokine identified in 2007,^46^ and several controversial reports exist regarding its signaling mechanisms and effects. Xue et al^47^ introduced chemerin as a proinflammatory adipokine in psoriatic arthritis patients. Also, an elevated level of serum chemerin in inflammatory and immunologic conditions, such as inflammatory bowel disease (IBD), Crohn’s disease, and ulcerative colitis, was reported in a study by Weigert et al.^48^

Huang et al^31^ reported that the chemerin level in synovial fluid is positively correlated with the deterioration severity of cartilage tissue in knee osteoarthritis, and it might act as an inflammatory factor by promoting the inflammatory signal pathway. Yamawaki et al^49^ treated vascular endothelial cells with different concentrations (1‒300 ng/mL) of chemerin and indicated that chemerin possibly has anti-inflammatory effects at low concentrations and proinflammatory properties at high concentrations. They suggested that the effect of chemerin depends on its concentration and, more importantly, the types of target cells. To the best of our knowledge, only one study has investigated chemerin levels in periodontal diseases,^35^ and there is no literature exploring this adipokine in diabetic patients or the effect of periodontal treatment. In the present study, the salivary concentration of chemerin decreased in response to periodontal treatment; however, this change was not significant. Since periodontitis is a chronic inflammatory state, and its treatment results in the resolution of inflammation, theoretically the amount of chemerin should have reduced. Nevertheless, the low concentration of this adipokine in saliva (than that of serum) causes the changes to be negligible.

In contrast, the study of Özcan et al^43^ considered chemerin a more specific biomarker than visfatin in differentiating periodontitis from gingivitis, indicating that salivary chemerin is a proper indicator of periodontitis.

This interventional study’s primary finding was that improvements in clinical parameters by periodontal treatment were followed by a significant reduction in the salivary concentration of progranulin in diabetic patients. Progranulin has been previously found to have anti-inflammatory effects against rheumatoid arthritis by preventing TNF-α from binding to its receptor.^50^ However, the role of progranulin in the inflammatory process is more complicated, and it seems that this adipokine is divided into a smaller peptide called granulin, which has proinflammatory effects and neutralizes the anti-inflammatory properties of progranulin. Therefore, based on the affected tissue, progranulin exerts its proinflammatory or anti-inflammatory effects. During inflammation in periodontitis, neutrophils and macrophages release serine proteases that break progranulins to granulin.^32^

Özcan et al^43^ showed that this parameter was higher in periodontitis patients than healthy individuals, and progranulin decreased significantly three months after treatment. In another study in 2015, the same researchers reported no significant differences in progranulin between patients with periodontitis or gingivitis, and healthy controls.^35^

Concerning clinical periodontal parameters, GI was positively correlated with visfatin, and a positive correlation was found between PPD and chemerin in the present study. Moreover, salivary progranulin was positively correlated with GI and PPD. However, no correlation was found between CAL and PI and any of the salivary adipokines. The positive relationship between the level of salivary adipokines and periodontal measures indicates their function in the pathogenesis of inflammation and periodontal disease.

The limitations of this study should be considered. First, the limited number of cases restrains the generalizability of our results; therefore, further clinical studies with a larger sample size are needed to validate the findings. Second, a control group was not included in the trial design because of ethical concerns and our limitation in recruiting eligible subjects.

## Conclusion

Non-surgical therapy in diabetic patients with periodontitis yielded good results in improving the clinical status of periodontitis, and improvements were observed in clinical indices of GI, PPD, and PI. However, the analysis of visfatin and chemerin adipokines in the present study did not reveal reliable data regarding the relationship of these adipokines with periodontitis, diabetes, and the effect of non-surgical therapy except for progranulin concentration which showed a significant decrease after periodontal treatment.

## Acknowledgments

Not applicable.

## Authors’ Contributions

NZ helped with funding acquisition and observing the clinical procedures. SG conceived the study and performed the scientific writing of the manuscript. MA provided the diabetic cases and helped with the scientific content of the manuscript. FA carried out the non-surgical periodontal treatment and pre- and post-treatment clinical examinations. MMA performed the ELISA and statistical analyses. All the authors have read and agreed to the published version of the manuscript.

## Funding

This investigation was approved as a research project, and funding was at least in part provided by the Research Institute of the Dental School, Kermanshah University of Medical Sciences.

## Competing Interests

The authors declare no competing interests with regards to the authorship and publication of this article.

## Ethics Approval

The research was reviewed and approved by Ethics Committee of Kermanshah University of Medical Sciences (IR.kums.rec.1395.564).
